# Can Natural Speech Prosody Distinguish Autism Spectrum Disorders? A Meta-Analysis

**DOI:** 10.3390/bs14020090

**Published:** 2024-01-26

**Authors:** Wen Ma, Lele Xu, Hao Zhang, Shurui Zhang

**Affiliations:** School of Foreign Languages and Literature, Shandong University, Jinan 250100, China; mawen@sdu.edu.cn (W.M.); 201900020037@mail.sdu.edu.cn (S.Z.)

**Keywords:** prosody, autism, machine learning, natural speech

## Abstract

Natural speech plays a pivotal role in communication and interactions between human beings. The prosody of natural speech, due to its high ecological validity and sensitivity, has been acoustically analyzed and more recently utilized in machine learning to identify individuals with autism spectrum disorders (ASDs). In this meta-analysis, we evaluated the findings of empirical studies on acoustic analysis and machine learning techniques to provide statistically supporting evidence for adopting natural speech prosody for ASD detection. Using a random-effects model, the results observed moderate-to-large pooled effect sizes for pitch-related parameters in distinguishing individuals with ASD from their typically developing (TD) counterparts. Specifically, the standardized mean difference (SMD) values for pitch mean, pitch range, pitch standard deviation, and pitch variability were 0.3528, 0.6744, 0.5735, and 0.5137, respectively. However, the differences between the two groups in temporal features could be unreliable, as the SMD values for duration and speech rate were only 0.0738 and −0.0547. Moderator analysis indicated task types were unlikely to influence the final results, whereas age groups showed a moderating role in pooling pitch range differences. Furthermore, promising accuracy rates on ASD identification were shown in our analysis of multivariate machine learning studies, indicating averaged sensitivity and specificity of 75.51% and 80.31%, respectively. In conclusion, these findings shed light on the efficacy of natural prosody in identifying ASD and offer insights for future investigations in this line of research.

## 1. Introduction

Speech prosody has a crucial role in social reciprocity, which can directly modify meanings in daily communication [1,2,3,4,5,6]. Individuals undergoing difficulties in communication commonly exhibit prosodic abnormalities, as a result of which atypical prosody can serve as a promising biomarker for neurodevelopmental disorders (NDDs) [6]. ASD, a specific NDD with spectrum features, is commonly co-morbid with other NDDs, such as intellectual disabilities or global developmental delay [7]. ASD is characterized by a dyad of impaired social communication as well as restricted and repetitive patterns of behaviors and interests [8,9]. For ASD patients, atypical prosody substantially contributes to their social oddness [10,11] and prominently impedes their social acceptance [11,12]. Therefore, prosodic disorders can be distinctive characteristics of ASD [3,8,13,14], which offers insights into the etiological understanding and fingerprint screening [15,16].

With high ecological validity and sensitivity, natural prosody has drawn cumulative research attention to portray the prosodic profile of the autistic population [4,17]. It has been demonstrated that prosodic features in natural contexts show representativeness and sensitivity in distinguishing individuals with ASD [13]. Employing the power of natural speech, machine learning is a burgeoning field that aims to identify ASD more efficiently [13,18,19,20]. However, previous literature has highlighted several challenges in this line of research, including the inconsistent description of autistic prosodic features, unaccounted between-study heterogeneity, and limited quantitative evidence on specific aspects of autistic prosody performance [21,22]. Therefore, this study performed a fine-grained meta-analysis to offer a comprehensive evaluation of the significance of natural speech prosody in the ASD population.

## 2. Literature Review

Prosodic disorders are inherently related to socio-communicative problems [11,22,23], which potentially provides insightful observations on the core symptomatology of ASD [15,16]. In speech communication, social information is encoded and conveyed via sound patterns [2]. However, autistic individuals commonly struggle to establish the cognitive foundation necessary for recognizing speech prosody [4], due to challenges in inferring communication intentions from speech [24] and/or the difficulties in integrating multi-channel processing [25]. Reciprocally, the production of atypical prosody is exaggerated by inaccurate perception because of the aberrant auditory system of ASD patients [26]. The impaired speech chain shows a long-lasting impact on the social-pragmatic ability of ASD children whose prosodic abnormality is resistant to the development of other language abilities [12,27]. Therefore, prosodic disorders have been recognized as key and early features of ASD [3,8,13,14].

Prosodic disorders of autistic individuals have prompted a fair amount of research attempts [16], but the general pattern of prosody cannot be characterized for ASD patients, considering the inconsistent findings among studies due to the large individual heterogeneity and measurement variability [12]. For example, despite the conventional description of “mono-toned” or “machine-like”, autistic speech has been reported to show a higher pitch variability with accumulating evidence [3,15,22,28]. The inconsistency is further complicated by a variety of factors moderating the pitch variability, including IQ, age, gender, autistic severity, and language capacity [3,16,29]. In the same vein, research has revealed discrepant results on the pitch mean. Several studies have reported higher values of mean pitch for ASD over TD, regardless of constrained or natural speech tasks [3] and across positive or negative emotional contexts [30]. However, other investigations have found non-significant differences in the mean F0 between ASD and TD, with a refined control/modulation of IQ and verbal ability across the two groups [1,16]. Furthermore, the temporal performances observed are also perplexing due to different criteria of duration [29] and large heterogeneity among individuals with ASD [1,31]. In conclusion, these insistent findings have highlighted the considerable heterogeneity in terms of autistic prosody [12,22]. This underscores the necessity for a fine-grained, sensitive, and explicit assessment of prosodic characteristics for monitoring, detecting, diagnosing, and treating ASD patients [10,16,32,33].

Despite the aforementioned inconsistencies, the analyses of natural prosody are supposed to provide a more accurate portrayal of autistic prosody [4]. It is noteworthy that prosodic features in natural speech have been demonstrated to be more representative and sensitive [13]. For example, accumulating studies have showed elevated pitch mean in natural speech for ASD patients, irrespective of tonal or non-tonal language speakers and across different age groups [28]. Furthermore, the pitch standard deviation of ASD groups is 82.6% larger than that of TD groups in a natural context [34]. With high sensitivity, prosodic characteristics of natural speech are incorporated in the clinical diagnosis of ASD [16,28]. For example, prosodic features or voice quality in natural speech were used in the Autism Diagnostic Interview-Revised and Autism Diagnostic Observation Schedule-in the Second Edition (the golden standards for the assessment of ASD) and Social Responsiveness Scale to detect autistic individuals [28,35]. In addition, aberrations of pitch variability in daily conversation are applied by clinicians to distinguish autistic speakers [3]. However, these conventionalized methods heavily rely on the accumulative knowledge and implicit experience of trained clinicians [17,36], with subject-dependent and time-consuming limitations. Therefore, the adoption of quantitative techniques has been advocated to advance ASD detection in clinical practices based on prosodic evidence [37,38,39].

There is a surging interest in employing machine learning algorithms trained by natural prosodic features for the automatic identification of ASD [13,18,19]. Specifically, a machine learning algorithm learns distributions and patterns from training data and then uses them to predict the target outcome [40]. Leveraging the power of the automated approach, a machine can achieve 67.6% accuracy even though the training data are remarkably limited (e.g., natural speech from four ASD children and four TD children) [20]. Importantly, with time-limited discourse clips (90 s per clip) and a larger sample size (20 ASD and 38 TD children), relatively more accurate results (70% accuracy) are obtained, suggesting encouraging results trained by limited data and better performances trained by larger data [13]. This finding has been confirmed by higher accuracy (more than 85%) with larger samples [5,39] and higher accuracy (88%) with a larger data corpus [41]. However, despite the promising accuracy of ASD detection using machine learning techniques, the inclusion of various acoustic parameters in different algorithms has led to remarkable variations in extant observations [39]. For instance, the algorithms trained by rhythm or by intonation relevant characteristics reached significantly different Area Under Curves (AUCs; e.g., 88.6%, 75%, and 55.9%) [5,42]. Therefore, these aforementioned findings have highlighted the promising prospects as well as the heterogeneity of machine learning in ASD detection.

Overall, natural speech prosody correlates with communicative development, which has received intense research interest for a better understanding and screening of ASD. However, the literature reviewed shows strikingly inconsistent discoveries regarding the distinctive characteristics of autistic prosody and the efficacy of machine learning in ASD detection. Performing a targeted meta-analysis may be helpful to deal with these inconsistencies and offer valuable insights for clinical practice. By aggregating eligible studies, a meta-analysis has the potential to mitigate the result bias of individual studies, provide reliable power with large sample sizes, find consistent patterns across studies, and offer invaluable insights for future empirical investigations and clinical interventions.

To the best of our knowledge, the two latest meta-analyses conducted by Fusaroli et al. [22] and Asghari et al. [21] examined the acoustic features of individuals with ASD. Fusaroli et al. evaluated prosodic and voice quality data derived from constrained, spontaneous production, or social interaction of autistic patients, which observed salient standard mean differences in pitch mean value, pitch range, and pitch variability between ASD and TD individuals. In addition to the univariate studies, Fusaroli et al. also revealed the encouraging accuracy of machine learning trained by prosodic features for ASD detection. Although the results were inspiring, the review by Fusaroli et al. [22] did not explore the specific performance of autistic prosody in different production conditions (i.e., constrained production, spontaneous production, and social interaction) or between different age groups. Moreover, their review of the machine learning outcome was restricted by the limited amounts of the included data. To verify the findings of Fusaroli et al. on the prosodic performance of ASD, Asghari et al. [21] conducted an updated review of univariate studies with more eligible data. Their findings replicated the significant differences between ASD and TD populations in terms of pitch mean, pitch range, and speech duration, but found non-significant differences between the two populations in pitch standard deviation and speech rate. Additionally, Asghari et al. classified the task types and age groups, which revealed that the confounding factors might have a significant moderating role in effect size pooling, such as task types in pooling pitch range and duration differences and age groups in intensity mean differences. However, caution should be exercised while interpreting the results of Asghari’s research. On the one hand, the limited samples of each subgroup for the moderator analysis could deviate from the precision of the final results. On the other hand, despite the refinement of task types, considerable heterogeneity resulting from different tasks remained apparent (e.g., *I*^2^ = 91.4% in a narration task, *I*^2^ = 80.7 in a conversation task).

## 3. The Present Study

This study was built upon the prior reviews for twofold expansions. Firstly, given the high ecological validity and sensitivity of natural prosody in evaluating the communicative ability of ASD patients, we focused on the analysis of prosodic features in spontaneous speech. The specification was intended to reduce heterogeneity, better profile autistic prosodic performance, and provide clinical implications for ASD diagnosis. Secondly, we updated the reviewing procedure to January 2024 and included comprehensive research across multiple databases. As a result, we extracted and coded a total of 25 eligible univariate studies on spontaneous speech and 18 multivariate studies on machine learning. By incorporating newly available evidence, this state-of-the-art review could add statistical power and provide valuable implications for the diagnosis and intervention of the autistic population.

Specifically, this study aimed to extend prior efforts to elucidate the between-study heterogeneity and the perplexing inconsistencies of the literature reviewed. Therefore, a meta-analysis of autistic prosodic performance, subgroup analysis, and machine learning analysis of model performance were conducted. Research questions for this study were raised as follows:

Q1: Can natural prosodic features differentiate ASD from TD groups?

Given the previous evidence that prosody was a reflection of social communicative ability [11,22,23] and ASD populations were deflected in theory of mind [24], we predicted that a large number of prosodic features might have conspicuous differences between the ASD and TD groups.

Q2: Are there confounding factors that affect size pooling?

Previous research has proved that large individual differences in the autistic group [1,31], including severity [1,22] and age [43], could influence the prosodic performance of ASD. Therefore, we predicted that there were potential moderators, such as age groups and task types, with a role in effect size pooling.

Q3: How do machine learning models trained by natural speech perform in ASD detection?

The previous systematic review by Fusaroli et al. [22] concluded a promising landscape of machine learning in ASD detection. In addition, more recent work conducted by Chi et al. [13] also showed that even limited data could train machine learning to detect ASD populations. Therefore, we predicted that machine learning might have promising accuracy, specificity, precision, and sensitivity in ASD detection.

## 4. Materials and Methods

### 4.1. Search Strategy

To identify the relevant articles, we conducted exhaustive literature research in the following databases: Biosis Previews, Elsevier Science-direct, Embase, Eric, Inspec, MEDLINE, PorQuest, Scopus, and Web of Science Core Collection from the time of their first publication to January 2024. The following combination of words was used as search terms: (a) “autism OR autistic OR ASD OR HFA OR Asperger OR PDD” AND (b) “prosody OR prosodic OR phonetics OR phonology OR phonological OR voice” AND (c) “rhyme OR spontaneous discourse OR conversation OR speech OR automatic OR melody OR natural conversation OR narration”. In addition, manual searches of reference lists were conducted to identify more potential eligible studies.

Furthermore, the identified studies eligible for inclusion in the review were screened with the following inclusion criteria: (1) Studies should include individuals who had a confirmed diagnosis of ASD with normal nonverbal intelligence and had no hearing or visual disorders. (2) Studies should have TD counterparts enrolled in a control group. (3) Studies should clearly report the detailed statistical data for effect size calculation, such as sample sizes, mean differences, standard deviation, AUC, recall, and precision. (4) Studies should employ experimental or quasi-experimental methods and have a detailed report on the quantitative research design. Additionally, studies had to be excluded for one of the following reasons: (1) The studies were meta-analyses or reviews without origin data. (2) The studies did not provide sufficient data to qualify the calculation of an effect size. (3) The studies did not employ natural speech tasks to elicit prosody data.

### 4.2. Risk of Bias Assessment

**Figure 1:**
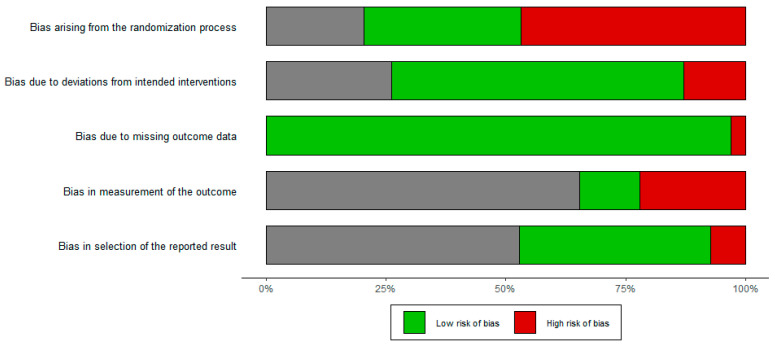
Results of risk of bias assessment.

The quality of the included data was evaluated using the Risk of Bias2 (ROB2) assessment tool in five fields: randomization process, intended interventions, data completeness, outcome measurement, and result reporting intactness. Two independent reviewers rated the reports, and any disagreements were resolved through discussion to reach a consensus. The results were visualized using a summary barplot figure, where the proportion of studies with a given risk of bias judgement in each ROB2 domain would be revealed (Figure 1). The risk of bias plot showed that bias due to deviations from intended interventions, missing outcome data, measurement of the outcome, and selection of the reported results was less likely to have high risk. However, the randomized process due to the selection of qualified participants might bias the final results.

### 4.3. Data Extraction

Data were extracted targeting the three research questions. Firstly, related statistical values for prosodic measures (e.g., sample sizes, means, standard deviations, *t* value, and *F* value) and numbers of participants were extracted for the calculation of effect sizes. In light of the findings in the literature, potential moderators (e.g., task types, speaking languages, and ages of participants) were coded. Thirdly, characteristics of machine algorithms were extracted, such as types of data, number of participants, and results of performance (i.e., AUC, accuracy (ACC), sensitivity (SENS), specificity (SPEC), and precision (PREC)).

In classifying task types, natural speech was defined as discourse that occurred without explicit elicitation [44] and exhibited acoustic distinctions from controlled or read speech [29,45]. Natural language, by virtue of approximating real-world social situations and having high ecological validity, formed the basis of linguistic communication [46] and portrayed social phenotypes of ASD [15]. There were three widely reported types of natural speech in the included research, namely narration (e.g., story-telling and picture-describing), conversation (e.g., question–answer tasks and semi-structured ADOS interviews), and interaction (e.g., free talk and game-playing) [20]. These three tasks exhibited varying degrees of spontaneity. For example, narration, while natural in nature, required great stability and differed from genuinely spontaneous [36]. Compared with narration, conversation relied on the shared social knowledge of interlocutors, occurring spontaneously and reciprocally [47]. Although conversation shared characteristics of interaction [34], the latter had a higher degree of social spontaneity and interpersonal dynamics in nature [22].

### 4.4. Statistical Analysis

Effect sizes for continuous variables were usually calculated as standardized mean differences with Cohend’s *d*, of which the magnitude was interpreted as a slight (0.2), medium (0.5), and large (0.8) effect. However, Cohend’s *d* could meet upward bias when the sample number of a study was limited (*n* < 20) [48]. Since the included studies varied in group sizes and the majority recruited a restricted number of participants, Hedges’ *g* was used to computerize effect sizes, which is appropriate for studies with limited sample sizes. To aggregate the effect sizes, we ran the meta-analysis under a random-effect model, considering that the true effect could be influenced by both the sampling error and between-study heterogeneity. To further control uncertainty regarding between-study heterogeneity, the meta-analysis was adjusted with the Knapp–Hartung adjustment.

We quantified variances in true effects using estimates of Tau^2^, which were run under a restricted maximum likelihood to avoid any bias from limited sample sizes. We assessed the between-study heterogeneity using *I*^2^, which showed the percentage of true variability in observed heterogeneity and was interpreted based on the thresholds of low (25%), moderate (50%), and high (75%) heterogeneity [49]. To further explain a specific heterogeneity pattern, a subgroup analysis was performed to explore specific confounding factors in effect size aggregation. For this process, we previously synthesized the sample sizes of ASD and TD groups, age groups, languages, severity of autism, task types of natural speech production, and available results of acoustic measures. Given that the confounding factors might collectively or interactively exert a moderating role, multi-model construction, inference, and interactions were conducted.

Through the above process, the meta-analytic techniques tried to reveal an unbiased estimate of the aggregated effect size. However, studies with unfavorable findings might be unpublished, and the pooled estimates were distorted due to the publication bias. Funnel plots, which can also evaluate the bias from small-study effects, were employed to assess the potential publication bias. Interpreting the results of funnel plots was to judge the plot asymmetry in a qualitative way, which was complemented by Egger’s test to testify to quantitative evidence [50].

The statistical analysis was performed in R 4.2.3 via the use of tidyverse v.2.0.0, meta v.6.2-1, metaphor v.4.0-0, dmetar 0.0.9, and robvis 0.3.0 packages.

## 5. Results

### 5.1. Study Selection Overview

**Figure 2:**
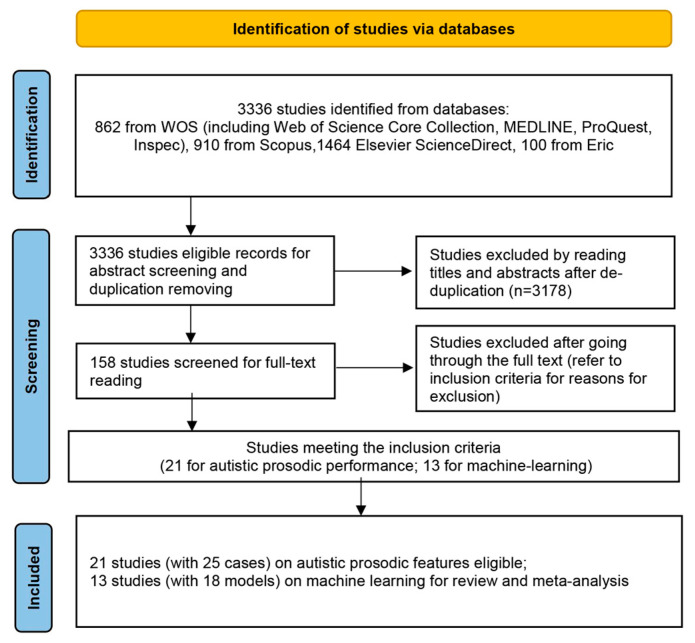
Flow diagram of the search procedure according to the PRISMA guidelines.

The research in electronic databases identified 3336 studies that were retained for title and abstract screening. After the removal of duplicates and other irrelevant studies, 158 full-text articles were evaluated. In total, 21 papers with 25 acoustic studies on autistic prosodic features and 13 papers with 18 studies on automatic machine learning were ultimately included (see Figure 2 for a description of the selection process). Table 1 provides a descriptive overview of the characteristics of studies on rhythmic features: the number of participants, the age (mean, standard deviation, and group) of participants, and the SMD of prosodic features, whereas Table 2 provides an overview of temporal features. A succinct overview of the included machine learning studies is outlined in Table 3.

### 5.2. Results of Prosodic Differences between ASD and TD Groups

#### 5.2.1. Pitch Mean

**Figure 3:**
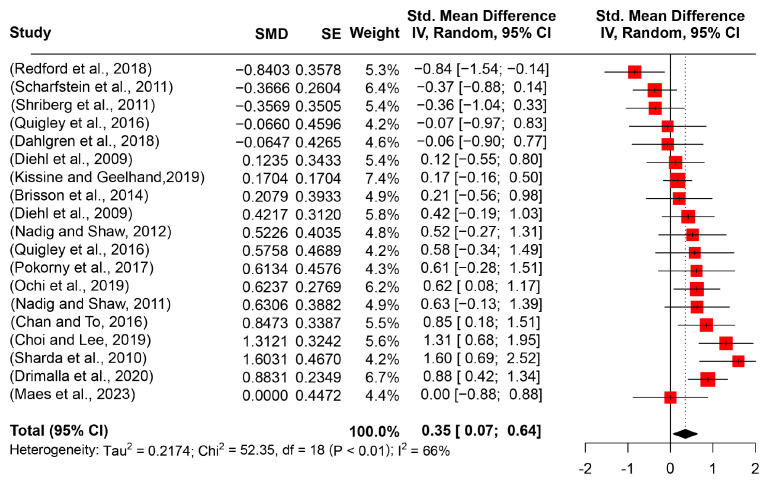
Forest plot for the meta-analysis of pitch mean differences [2,3,11,16,17,29,31,34,36,51,52,53,54,55,56,57].

Pitch is generated by pharynx vibration and reflects the frequency of voice. In the review, 19 articles with 20 studies (416 participants with ASD and 351 TD counterparts) investigated the difference in mean pitch value between the ASD and TD groups. After synthesizing the 18 experimental cases in a meta-analysis, the pooled effect size was 0.3528 (95%CI [0.0698 0.6358], *I*^2^ = 65.6%) (see Figure 3). Given the zero-exclusive 95% confidence interval and the significant results of the *t*-test (*t* = 2.59, *p* = 0.0181), the larger mean pitch value of ASD was remarkable. Furthermore, neither moderators (age groups and task types) nor their multi-regressive or interactive roles significantly functioned in the pooled effect size.

#### 5.2.2. Pitch Range

**Figure 4:**
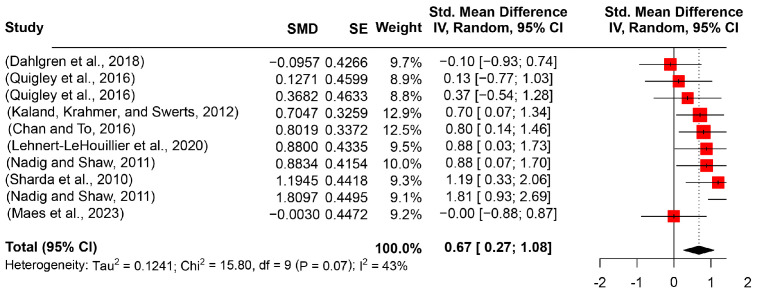
Forest plot for the meta-analysis of pitch range differences [3,29,52,54,56,57,58,59].

The pitch range indicates the scope of changes in pitch and is calculated by the max-min differences. In this review, 10 studies (137 participants with ASD and 124 TD participants) were included. In terms of the pooling results from the studies, the achieved effect size was significantly large (SMD = 0.6744, 95%CI [0.2698, 1.0790], *I*^2^ = 43%) (see Figure 4). This large effect was evidenced by statistical estimates of the *t*-test (*t* = 3.77, *p* = 0.0044). Further subgroup analysis indicated that the moderating role of age groups was substantial (*t* = 2, *p* = 0.0005).

#### 5.2.3. Pitch Standard Deviation

**Figure 5:**
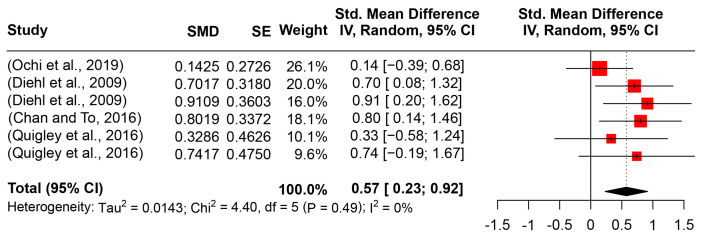
Forest plot for the meta-analysis of pitch SD differences [16,34,52,54].

Different from the pitch range, the pitch standard deviation reflects the dispersion degree of the pitch value. With the six eligible studies (142 ASD participants and 92 counterparts), the pooled effect size reached a significantly strong effect (SMD = 0.5735, 95%CI [0.2350, 0.9135], *I*^2^ = 0) (see Figure 5). Considering the non-zero overlapped confidence interval and zero reported between-heterogeneity, the larger pitch standard deviation of ASD groups was salient. Owing to the limited number of included studies, confounding factor analysis in the meta-analysis concerned with pitch SD was not performed.

#### 5.2.4. Pitch Variability

**Figure 6:**
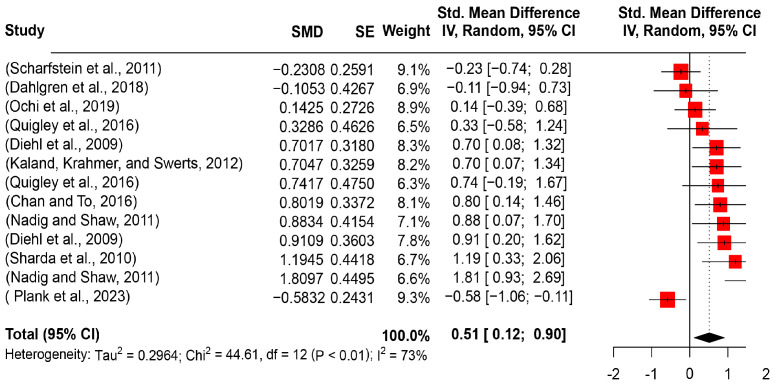
Forest plot for the meta-analysis of pitch variability differences [3,16,29,34,51,52,54,56,58,60].

Although the pitch range and pitch standard deviation indicate changes in pitch, their combination convincingly indicates the magnitude of pitch variability [28]. In the review, a total of 13 experimental studies (274 ASD participants and 241 TD participants) were included. After aggregating the results, a significantly large effect size was revealed (SMD = 0.5137, 95%CI [0.1237, 0.9037], *I*^2^ = 73.1%) (see Figure 6). The significant effect size was statistically evidenced (*t* = 4.27, *p* = 0.0141). A further confounding factor analysis revealed that no task type, age group, or interactive model had a moderating role.

#### 5.2.5. Utterance Duration

**Figure 7:**
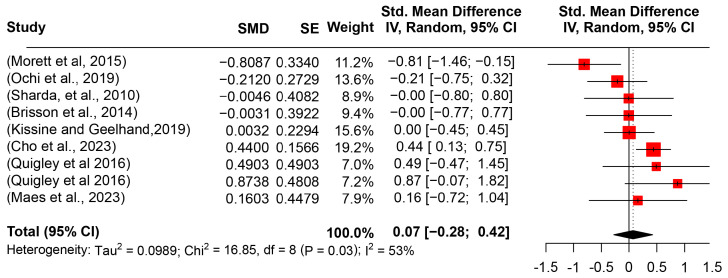
Forest plot for the meta-analysis of utterance duration differences [15,31,34,36,37,52,56,61].

Utterance duration was investigated by nine studies in the review (224 participants with ASD and 174 TD counterparts). Synthesizing the studies in the meta-analysis, the mean difference between ASD and TD groups was slight (SMD = 0.0738, 95%CI [−0.2768, 0.4244], *I*^2^ = 52.5%) (see Figure 7), which was also observed in the *t*-test (*t* = 0,49, *p* = 0.6404). Further moderator analysis of the two confounding factors (age groups and task types) indicated no significant moderating or interactive role.

#### 5.2.6. Speaking Rate

**Figure 8:**
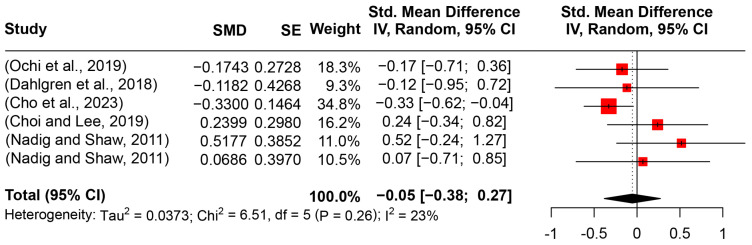
Forest plot for the meta-analysis of speaking rate differences [3,15,29,34,55].

Six papers on the speaking rate (158 participants with ASD and 133 TD participants) were included in the review. After pooling the findings of the studies, the meta-analysis reported a small effect size (SMD = −0.0547, 95%CI [−0.3818, 0.2725], *I*^2^ = 23.2%) (see Figure 8). However, the standardized mean difference was insignificant (*t* = −0.43, *p* = 0.6855). Given that only six studies were included in the meta-analysis, further moderator analysis and model construction were neglected.

#### 5.2.7. Intensity Mean and Variation

Intensity quantifies the energy of sound waves and influences information delivery in speech communication. The intensity differences between ASD and TD groups were investigated by six eligible studies in this review. Specifically, four available studies reported the intensity mean differences between ASD and TD, and all but one revealed a higher intensity mean of the ASD groups than TD ones. For intensity variability, only two eligible studies were included in the review. Although the two studies agreed that ASD groups had higher intensity variability, the degrees of the difference they indicated were completely different. Ochi et al. [34] examined the intensity of high-functioning autistic people (HFA) in their semi-structured conversation and revealed slight intensity standard deviation differences between ASD and TD (SMD = 0.1275, SD = 0.2726). On the contrary, Choi and Lee [55] found that in the interaction and communication speech of HFA, the difference could reach a large effect (SMD = 0.998, SD = 0.3131). Owing to the limited number (*n* < 6) of included papers, a meta-analysis failed to run.

### 5.3. Results from Machine Learning for ASD Diagnosis

**Figure 9:**
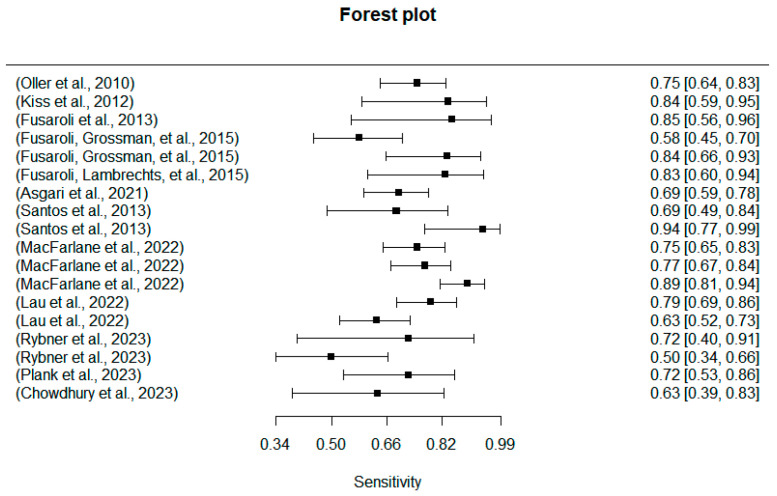
Forest plot of the machine’s sensitivity [5,19,32,39,42,60,62,63,64,65,66,67].

**Figure 10:**
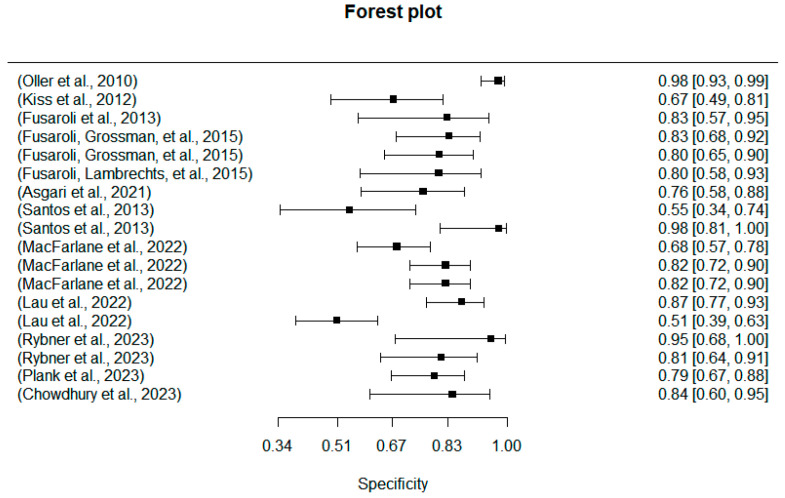
Forest plot of the machines’ specificity [5,19,32,39,42,60,62,63,64,65,66,67].

The previous section reviewed the prosodic patterns of the ASD group and revealed their prosodic differences from the TD group. In this section, a second set of 18 studies (see Table 3) about machine learning were evaluated. Machine learning studies, different from the univariate ones focusing on specific prosodic feature(s), seek to train multiple datasets to automatically identify the ASD populations. With regard to results, all but two multivariate studies in the review reached above 70% and up to 98% accuracy. A more detailed overview of the specificities and sensitives of the machine learning studies was reported in Figure 9 and Figure 10, of which the averaged specificities and sensitives achieved 75.51% and 80.31%.

In the machine learning process, four steps were typically involved, namely data extraction, selection, classification, and validation. The first process involved the extraction of voice features from the speech recordings. The extracted acoustic features had significant overlaps with those discussed in the previous section (e.g., mean and standard deviation of pitch, duration, and intensity), but also included additional parameters like harmonic-to-noise ratio (HNR), Mel-frequency cepstral coefficients (MFCC), jitter, and shimmer [13,18,19,67]. Considering that the extracted data was likely to be redundant, it was necessary to reduce overfit potentiality and promote the efficiency of machine learning algorithms. Therefore, features with remarkable contributions to distinguish ASD from TD groups were selected with tools like correlation analysis [53,67], principal component analysis, factor analysis [18,62], ElasticNet [63,64,65], and Geneva Minimalistic Acoustic Parameter Set (GeMAPS) [68]. Data selection was further classified by tools such as native Bayed (NB) [42], support vector machines (SVMs) [5,20,41,60,66,68], probabilistic neural networks (PNNs) [19], speech-related vocal islands (SVIs) [62], or random forests [67]. Since machine learning was not merely to find a model explaining the current data but to create a model that generalizing to new data [69]. To ensure generation for out-of-data testing, cross-validation (CV) [66,67] was frequently reported, with 5-fold CV [32,63,64,65,66], 10-fold CV [5,19], and leave-out procedures [19,39,42,67]. For a more comprehensive introduction and overview of multivariate machine learning processes, please see books written by Bishop [70] and Hastie et al. [71].

### 5.4. Publication Bias and Risk of Bias

Publication bias was evaluated using funnel plots and Egger’s and Begg’s tests. The results showed that the funnel plots of the meta-analyses aforementioned (except the plot of pitch variability) appeared to have a systematic distribution, and Egger’s tests obtained *p*-values larger than 0.05. These results indicated that the review had low risks of publication bias.

## 6. Discussion

With high ecological sensitivity and validity, natural speech prosody has gained considerable research attention for identifying autistic individuals. Through the aggregation of relevant research, the present study showed that (1) pitch-related features had significantly differential power between ASD and TD groups, whereas the power of temporal features was non-significant; (2) different task types could have no significant role, while the pitch range performance of individuals with ASD could be influenced by age groups; and (3) machine learning trained by natural speech samples showed encouraging accuracy in ASD detection, with an averaged sensitivity and specificity of 75.51% and 80.30%, respectively. To our knowledge, this study represents the first meta-analysis that focuses on the power of natural prosody in quantitatively assessing and automatically identifying ASD populations. These findings have highlighted the potential of natural speech prosody for high-efficient monitoring, detection, and intervention in individuals with autism, pointing to a promising direction for future research.

### 6.1. Prosodic Performance of ASD

In this review, the aggregated standardized mean difference for pitch value between ASD and TD achieved a positive medium effect (SMD = 0.3528, 95%CI [0.0698, 0.6358]). The pooled mean differences were significant between the two groups for pitch variability (SMD = 0.5137, 95%CI [0.1237, 0.9037]), pitch range (SMD = 0.6744, 95%CI [0.2698, 1.0790]), and pitch standard deviation (SMD = 0.5735, 95%CI [0.2320, 0.9150]). Notably, the zero-exclusive confidential interval along with the low-to-moderate heterogeneity revealed the robustness of melodic differences in autistic speech. The findings were in line with the previous literature [3,30,54], which consolidated the significance of pitch-related features in distinguishing ASD [32].

The abnormal pitch-related features observed in autistic individuals could be attributed to the deviated speech chain [25]. Speech is generated by the vibration of vocal cords, but the source–filter theory [72] has indicated the atypical vocal cords of autistic people [73], which can lead to different speech production. Speech sounds can be aberrantly perceived by ASD patients [26], which may directly impact the perception-production loop. Furthermore, autistic individuals tend to have difficulty inferring pragmatic or mental information from natural speech [4], due to their deficits in theory of mind [24]. Therefore, the impaired ability to perceive and process speech sounds shows potential to explain and reflect the atypical production performance of autistic children.

However, contrary to the findings of the current review, several studies observed non-significant differences in the mean F0 between the ASD and TD groups. For example, non-significant results were found when participants with HFA were included [16] or confounding variables (e.g., full-scale IQ) were controlled [1]. The selective criteria can deviate from the ecological validity of autistic natural prosody performance, potentially influencing the results. In addition, the selection can be a manifestation of the spectrum dimension of autistic patients, who show a wide range of degrees in social communication ability. The heterogeneity is evident in the overlapping pitch differences between ASD and TD groups [54], as well as the high pitch variations within the ASD group [16]. This has highlighted the necessity of considering individual heterogeneity when confirming the pitch performance of the autistic population. In addition to participant heterogeneity, between-study differences can also contribute to non-significant pitch-related differences between ASD and TD groups. A recent study on Mandarin-speaking autistic children indicated that the differences in pitch performances between ASD and non-ASD groups could be language-dependent issues since the differences were reported to be non-significant in native English speakers [1,3,16]. However, by pooling the results from participants with a large heterogeneity and multi-language speaking backgrounds, the current research has highlighted the significant pitch-related differences between ASD and TD groups, which can draw alerts to the general power of pitch features in ASD detection.

Moreover, the review showed slightly negative mean differences in speech rate (SMD = −0.0547, 95%CI [−0.3818, 0.2725]) and in speech duration (SMD = 0.0738, 95%CI [−0.2768, 0.4244]) between ASD and TD groups. With the confidential intervals containing zero, the differences were non-significant, indicating the instability of using temporal features to detect ASD groups. The non-significant difference was consistent with previous meta-analyses [21], raising doubts about the distinctive power of the autistic speech rate. There are several possible explanations for these non-significant differences in temporal performance between the two groups. Firstly, autistic speakers may perceive daily communication as a stressful task due to its high social knowledge and pragmatic demands [11]. Stuck by the increased load [74], autistic children tend to produce shorter utterances [31,61,75], and fewer non-grammatical pauses [12,75], reflecting the weak communicative activity of autistic individuals. Secondly, the lack of significant differences in temporal terms may also be attributed to heterogeneity in materials, measurement techniques, language, and individual differences [45]. Natural speech is characterized by remarkable variations [45]. With regard to types of speech, articulatory rate can differ between spontaneous speech, connected read speech, and sentence reading [76]. Additionally, large individual differences within the autistic patients [1,31], including severity [1,22] and age [43], have also been widely observed to contribute to their varied temporal performance. Furthermore, heterogeneity may exist across different studies conducted by diverse researchers, who may define an utterance as speech delimited by periods of silence or define it based on pragmatic or syntactic features [29].

However, unlike the current research, significant temporal differences between the ASD and TD groups have been reported in either univariate research or previous systematic reviews [21,22]. Notably, longer speech duration has also been long reported [77,78] and utilized as a diagnostic criterion for ASD [79]. Inherently, speech prosody functions as a bridge between verbal behaviors and mental disorders [25], and temporal performance is essentially a reflection of psychological phenomena [80]. The slower speed at which autistic people speak can be negatively correlated with their deflected social performance and therefore with the greater severity of ASD [1], which highlights the influence of individual differences in autistic speech. However, the current research focused on the natural prosody produced by a large number of autistic participants, encompassing a wide range of severity and heterogeneity, and found the aggregated temporal differences to be insignificant. This finding can implicate re-consideration when utilizing temporal features in ASD detection in the future.

### 6.2. Moderator and Heterogeneity Analysis

The current research revealed that all of the heterogeneity in the pooled effect size reached a low-to-medium (from 0% to 73.1%) degree, indicating an improvement in comparison with previous meta-analysis studies by Fusaroli [22] and Asghari [21]. The reduction in heterogeneity supports the potential reliability and stability of natural prosody in characterizing autistic people [4,81]. Specifically, different age groups, languages, severity of autism, and task types of natural production were coded as potential confounding factors in this study. It should be noted that the task type (i.e., narration, conversation, and interaction) was not a significant moderator in any effect size pooling, which contradicted the findings of the previous meta-analysis [21]. The differences could further highlight the sensitive and stable characteristics of natural prosody in autistic detection [13,17]. In addition, the current results showed that age groups (i.e., infants, children, and adults) had no salient moderating role in all of the meta-analysis except in pooling pitch range differences. However, the moderating power of age groups in pitch range differences could be deviated by the limited number of included studies (*n* = 10), warranting prudence in converging age groups while evaluating autistic prosodic performance. Furthermore, due to the lack of sufficient eligible data, some factors (e.g., languages and severity of autism) could not be incorporated in moderator analysis, which encouraged more open data in future research.

### 6.3. Predictive Value of Machine Learning

Synthesizing the multivariate machine learning studies, the averaged values of accuracy, sensitivity, and specificity were 77.96%, 75.51%, and 80.31%, respectively. Current findings reaffirm the promising results for automatic analysis in ASD detection [22]. Furthermore, leveraging high efficiency and non-invasion, machine learning techniques can complement the descriptive findings of clinicians and researchers [5]. This highlights the feasibility of adopting objective evaluations on prosodic parameters to identify individuals with autism or language delay [18,20,62].

However, regardless of the encouraging predictive value of machine learning techniques, the distinctive characteristics of natural prosody remain inconclusive for accurately identifying individuals with ASD [22,39]. For one thing, spontaneous speech recognition within the field of automatic modeling has only been in the initial phrase [45]. The limited number of related studies restricted a more refined meta-analysis to pool the distinctive performance of specific prosody. In addition, though previous research has indicated that different age groups (i.e., infants and adults) and different degrees of autistic severity can significantly influence machine learning results, their efforts on individual heterogeneity in machine learning performance have been largely overlooked. For another, no general attempt has been made to replicate findings across multiple studies [22]. The heterogeneity of machine learning procedures in different multivariate studies made it challenging to aggregate an estimate. Future studies call for more collaborative and open-research programs within the field of machine learning [22,37].

### 6.4. Implications and Limitations

The findings of this study are compatible with the view of previous meta-analyses that the panorama of autistic prosodic performance remains perplexing [22] and heterogeneous [21]. Focusing on the natural prosody, the present research takes a precise and further step to provide fine-grained and exhaustive evidence for the prosodic profile of ASD. This precision and renewal can provide a plausible direction for a better understanding of autistic prosody performance and can warrant more attention to the role of natural prosody in ASD detection. In addition, the moderating effect of age differences in autistic pitch range performance highlights the need to consider this factor in investigating autistic prosodic features and encourages exploring other confounding factors, such as the severity of autism, in future research.

Moreover, the current findings could have clinical implications for more efficient and objective screening and intervention for ASD populations. Firstly, previous research on autistic prosody has been hindered by the heterogeneity of tasks and reliance on qualitative analysis, contributing to obstacles to drawing plausible conclusions [22]. The present meta-analysis aimed to bridge the research gap by focusing on the natural speech context to reduce heterogeneity and pooling pure quantitative results to minimize subjective bias. Natural speech tasks are expected to complement qualitative analysis and inspire more participant-friendly approaches in order to improve assessment success and expand screening for individuals with ASD. Secondly, the incorporation of natural speech prosody in machine learning has revealed the feasibility of prosodic features as a promising marker for individuals with autism. Therefore, in the future, natural prosodic features can play a pivotal role in the efficient, objective, and reliable detection of ASD.

However, several limitations should be acknowledged for this review. Firstly, it was common for a single study to correspond to more than one effect size in our meta-analysis, especially for studies including multiple autistic groups or measuring multiple tasks. This could present a unit-of-analysis problem, potentially resulting in the double-counting of data. Secondly, due to the limited number of eligible data points, the meta-analysis cannot analyze some moderators, such as the severity of autism. In addition, though the moderators as task types and age groups were evaluated, their moderating role might be influenced by small sample sizes, calling for more precise and specified research in the future. Thirdly, inadequate reporting of statistical estimates prevented a thorough examination of the performance of specific prosodic features in automatically identifying ASD groups. In future research, more open and collaborative efforts are expected to be made.

## 7. Conclusions

Natural speech inherently indicates social communicative ability, serving as a potential biomarker for detecting ASD patients, who are typically characterized by socio-communicative disorders. To assess the efficacy of natural prosody in ASD detection, the present study conducted a meta-analysis on the prosodic differences between ASD and TD groups, a moderator analysis of between-study heterogeneity, and an investigation of the pertinent machine learning performance. The results have indicated that pitch-related features can significantly distinguish individuals with ASD from TD individuals. For the moderator analysis, different task types exert a slight influence on heterogeneity. Furthermore, natural prosody has shown promising accuracy in machine learning models for ASD detection. In a nutshell, the current research provides updated and fine-grained evidence for distinctive characteristics of autistic prosody, which corroborates the feasibility of natural prosody in ASD identification and offers a focused direction for future research.

## Figures and Tables

**Table 1:** Summary of pitch characteristics of ASD and TD in included studies.

Name	N_ASD	N_TD	Age_ASD	Age_TD	Group	Language	Task	PitchMeanASDvsTD
(Redford et al., 2018) [2]	17	17	M: 9 (Yr.) SD: 18 (mon.)	M: 8.9 (Yr.) SD: 15 (mon.)	Children	English	Conversation	−0.8403 (0.3578)
(Scharfstein et al., 2011) [51]	30	30	M: 0.57 (mon.)	M: 10.60 (mon.)	Children	English	Interaction	−0.3666 (0.2604)
(Shriberg et al., 2011) [11]	46	10	M: 69.9 (mon.) SD: 14.4 (mon.)	Range: 4–7 (Yr.)	Children	English	Conversation	−0.3569 (0.3505)
(Quigley et al., 2016) [52]	10	9	M: 12.12 (mon.) SD: 0.89 (mon.)	M: 11.95 (mon.) SD: 0.84 (mon.)	Infant	English	Interaction	−0.066 (0.4596)
(Dahlgren et al., 2018) [29]	11	11	M: 11.1 (Yr.) SD: 1.10 (Yr.)	M: 11.1 (Yr.) SD: 0.47 (Yr.)	Children	Swedish	Narration	−0.0647 (0.4265)
(Diehl et al., 2009) [16]	17	17	M: 8.81 (Yr.) SD: 2.13 (Yr.)	M: 9.49 (Yr.) SD: 2.22 (Yr.)	Children	English	Narration	0.1235 (0.3433)
(Kissine and Geelhand, 2019) [36]	38	38	M: 28.1 (Yr.) SD: 11.48 (Yr.)	M: 27.9 (Yr.) SD: 11.53 (Yr.)	NA	French	NA	0.1704 (0.1704)
(Brisson et al., 2014) [31]	13	13	M: 4.38 (mon.) SD: 0.88 (mon.)	M: 3.71 (mon.) SD: 1.39 (mon.)	Infant	French	Interaction	0.2079 (0.3933)
(Diehl et al., 2009) [16]	21	21	M: 13.58 (Yr.) SD: 2.10 (Yr.)	M: 13.24 (Yr.) SD: 2.09 (Yr.)	Children	English	Narration	0.4217 (0.312)
(Nadig and Shaw, 2012) [3]	15	11	M: 10.6 (Yr.) SD: 17 (mon.)	M: 10.8 (Yr.) SD: 23 (mon.)	Children	English	Interaction	0.5226 (0.4035)
(Quigley et al., 2016) [52]	10	9	M: 18.27 (mon.) SD: 0.85 (mon.)	M: 18.13 (mon.) SD: 0.88 (mon.)	Infant	English	Interaction	0.5758 (0.4689)
(Pokorny et al., 2017) [53]	10	10	NA	NA	Infant	Swedish	Interaction	0.6134 (0.4576)
(Ochi et al., 2019) [34]	62	17	M: 26.9 (Yr.) SD: 7.0 (Yr.)	M: 29.6 (Yr.) SD: 7.0 (Yr.)	Adult	Japanese	Interaction	0.6237 (0.2769)
(Nadig and Shaw, 2012) [3]	15	13	M: 11.0 (Yr.) SD: 19 (mon.)	M: 11.0 (Yr.) SD: 24 (mon.)	Children	English	Conversation	0.6306 (0.3882)
(Chan and To, 2016) [54]	19	19	M: 25.72 (Yr.) SD: 3.63 (Yr.)	M: 25.50 (Yr.) SD: 3.21 (Yr.)	Adult	Chinese	Narration	0.8473 (0.3387)
(Choi and Lee, 2019) [55]	17	34	M: 98.8 (mon.) SD: 18.6 (mon.)	M: 99.3 (mon.) SD: 20.7 (mon.)	Children	Korean	Conversation	1.3121 (0.3242)
(Sharda et al., 2010) [56]	15	10	M: 6.25 (Yr.) SD: 1.5 (Yr.)	M: 7.3 (Yr.) SD: 2.0 (Yr.)	Children	English-Hindi bilingual	Interaction	1.6031 (0.4670)
(Drimalla et al., 2020) [17]	37	43	M: 36.89 (Yr.)	M: 33.14 (Yr.)	Adult	German	Interaction	0.8831 (0.2349)
(Maes et al., 2023) [57]	10	10	M: 4 (Yr.); 06.9 (mon.) SD: 1 (Yr); 00.23 (mon)	M: 4 (Yr); 06.54 (mon.) SD: 0 (Yr); 09.82 (mon.)	Children	French	Interaction	0 (0.4472)
**Name**	**N_ASD**	**N_TD**	**Age_ASD**	**Age_TD**	**Group**	**Language**	**Task**	**PitchRangeASDvsTD**
(Dahlgren et al., 2018) [29]	11	11	M: 11.1 (Yr.) SD: 1.10 (Yr.)	M: 11.1 (Yr.) SD: 0.47 (Yr.)	Children	Swedish	Narration	−0.0957 (0.4266)
(Quigley et al., 2016) [52]	10	9	M: 2.12 (mon.) SD: 0.89 (mon.)	M: 1.95 (mon.) SD: 0.84 (mon.)	Infant	English	Interaction	0.1271 (0.4599)
(Quigley et al. 2016) [52]	10	9	M: 8.27 (mon.) SD: 0.85 (mon.)	M: 8.13 (mon.) SD: 0.88 (mon.)	Infant	English	Interaction	0.3682 (0.4633)
(Kaland, Krahmer, and Swerts, 2012) [58]	20	20	M: 28.9 (Yr.)	NA	Adult	Dutch	Interaction	0.7047 (0.3259)
(Chan and To, 2016) [54]	19	19	M: 25.72 (Yr.) SD: 3.63 (Yr.)	M: 25.50 (Yr.) SD: 3.21 (Yr.)	Adult	Chinese	Narration	0.8019 (0.3372)
(Lehnert-LeHouillier et al., 2020) [59]	12	12	M: 12.14 (Yr.) SD: 1.84 (Yr.)	M: 12.23 (Yr.) SD: 1.89 (Yr.)	Children	English	Conversation	0.88 (0.4335)
(Nadig and Shaw, 2012) [3]	15	11	M: 10.6 (Yr.) SD: 17 (mon.)	M: 10.8 (Yr.) SD: 23 (mon.)	Children	NA	Interaction	0.8834 (0.4154)
(Shardaet al., 2010) [56]	15	10	M: 6.25 (Yr.) SD: 1.5 (Yr.)	M: 7.3 (Yr.) SD: 2.0 (Yr.)	Children	English-Hindi bilingual	Interaction	1.1945 (0.4418)
(Nadig and Shaw, 2012) [3]	15	13	M: 11.0 (Yr.) SD: 19 (mon.)	M: 11.0 (Yr.) SD: 24 (mon.)	Children	NA	Conversation	1.8097 (0.4495)
(Maes et al., 2023) [57]	10	10	M: 4; 06.9 (Yr.) SD: 1; 00.23 (Yr.)	M: 4; 06.54 (Yr.) SD: 0; 09.82 (Yr.)	Children	French	Interaction	−0.003 (0.4472)
**Name**	**N_ASD**	**N_TD**	**Age_ASD**	**Age_TD**	**Group**	**Language**	**Task**	**PitchSDASDvsTD**
(Ochi et al., 2019) [34]	65	17	M: 26.9 (Yr.) SD: 7.0 (Yr.)	M: 29.6 (Yr.) SD: 7.0 (Yr.)	Adult	NA	Interaction	0.1425 (0.2726)
(Diehl et al., 2009) [16]	21	21	M: 13.58 (Yr.) SD: 2.10 (Yr.)	M: 13.24 (Yr.) SD: 2.09 (Yr.)	Children	English	Narration	0.7017 (0.318)
(Diehl et al., 2009) [16]	17	17	M: 8.81 (Yr.) SD: 2.13 (Yr.)	M: 9.49 (Yr.) SD: 2.22 (Yr.)	Children	English	Narration	0.9109 (0.3603)
(Chan and To, 2016) [54]	19	19	M: 25.72 (Yr.) SD: 3.63 (Yr.)	M: 25.50 (Yr.) SD: 3.21 (Yr.)	Adult	Chinese	Narration	0.8019 (0.3372)
(Quigley et al., 2016) [52]	10	9	M: 2.12 (mon.) SD: 0.89 (mon.)	M: 1.95 (mon.) SD: 0.84,mon.)	Infant	English	Interaction	0.3286 (0.4626)
(Quigley et al., 2016) [52]	10	9	M: 8.27 (mon.) SD: 0.85 (mon.)	M: 8.13 (mon.) SD: 0.88 (mon.)	Infant	English	Interaction	0.7417 (0.475)
**Name**	**N_ASD**	**N_TD**	**Age_ASD**	**Age_TD**	**Group**	**Language**	**Task**	**PitchVarASDvsTD**
(Scharfstein et al., 2011) [51]	30	30	M: 10.57 (Yr.)	M: 10.60 (Yr.)	Children	English	Interaction	−0.2308 (0.2591)
(Dahlgren et al., 2018) [29]	11	11	M: 11.1 (Yr.) SD: 1.10 (Yr.)	M: 11.1 (Yr.) SD: 0.47 (Yr.)	Children	Swedish	Narration	−0.1053 (0.4267)
(Ochi et al., 2019) [34]	65	17	M: 26.9 (Yr.) SD: 7.0 (Yr.)	M: 29.6 (Yr.) SD: 7.0 (Yr.)	Adult	NA	Interaction	0.1425 (0.2726)
(Quigley et al., 2016) [52]	10	9	M: 2.12 (mon.) SD: 0.89 (mon.)	M: 1.95 (mon.) SD: 0.84 (mon.)	Infant	English	Interaction	0.3286 (0.4626)
(Diehl et al., 2009) [16]	21	21	M: 13.58 (Yr.) SD: 2.10 (Yr.)	M: 13.24 (Yr.) SD: 2.09 (Yr.)	Children	English	Narration	0.7017 (0.318)
(Kaland, Krahmer, and Swerts, 2012) [58]	20	20	M: 28.9 (Yr.)	NA	Adult	NA	Interaction	0.7047 (0.3259)
(Quigley et al., 2016) [52]	10	9	M: 8.27 (mon.), SD: 85 (mon.)	M: 8.13 (mon.) SD: 8 (mon.)	Infant	English	Interaction	0.7417 (0.475)
(Chan and To, 2016) [54]	19	19	M: 25.72 (Yr.) SD: 3.63 (Yr.)	M: 25.50 (Yr.) SD: 3.21 (Yr.)	Adult	Chinese	Narration	0.8019 (0.3372)
(Nadig and Shaw, 2012) [3]	15	11	M: 10.6 (Yr.) SD: 17 (mon.)	M: 10.8 (Yr.) SD: 23 (mon.)	Children	NA	Interaction	0.8834 (0.4154)
(Diehl et al., 2009) [16]	17	17	M: 8.81 (Yr.) SD: 2.13 (Yr.)	M: 9.49 (Yr.) SD: 2.22 (Yr.)	Children	English	Narration	0.9109 (0.3603)
(Sharda et al., 2010) [56]	15	10	M: 6.25 (Yr.) SD: 1.5 (Yr.)	M: 7.3 (Yr.) SD: 2.0 (Yr.)	Children	English-Hindi bilingual	Conversation	1.1945 (0.4418)
(Nadig and Shaw, 2012) [3]	15	13	M: 11.0 (Yr.) SD: 19 (mon.)	M: 11.0 (Yr.) SD: 2 (mon.)	Children	NA	Conversation	1.8097 (0.4495)
(Plank et al., 2023) [60]	26	54	M: 34.85 (Yr.) SD: 12.01 (Yr.)	M: 30.80 (Yr.) SD: 10.42 (Yr.)	Adult	German	Conversation	−0.5832 (0.2431)

**Table 2:** Studies involving acoustic measures of duration or speech rate in ASD.

Name	N_ASD	N_TD	Age_ASD	Age_TD	Group	Language	Task	DurationASDvsTD
(Morett et al. 2015) [61]	18	21	M: 15.17 SD: 2.75	M: 15.81 SD: 2.42	Children	English	Narration	−0.8087 (0.334)
(Ochi et al., 2019) [34]	65	17	M: 26.9 (Yr.) SD: 7.0 (Yr.)	M: 29.6 (Yr.) SD: 7.0 (Yr.)	Adult	Japanese	Interaction	−0.212 (0.2729)
(Sharda, et al., 2010) [56]	15	10	M: 6.25 (Yr.) SD: 1.5 (Yr.)	M: 7.3 (Yr.) SD: 2.0 (Yr.)	Children	English-Hindi bilingual	Interaction	−0.0046 (0.4082)
(Brisson et al., 2014) [31]	13	13	M: 4.38 SD: 0.88	M: 3.71 SD: 1.39	Infant	French	Interaction	−0.0031 (0.3922)
(Kissine and Geelhand, 2019) [36]	38	38	M: 28.1 SD: 11.48	M: 27.9 SD: 11.5	NA	French	NA	0.0032 (0.2294)
(Cho et al., 2023) [15]	45	47	M: 25.7 (mon.) SD: 3.63 (mon.)	M: 25.5 (mon.) SD: 3.21 (mon.)	Children	Chinese	Conversation	0.44 (0.1566)
(Quigley et al. 2016) [52]	10	9	M: 2.12 (mon.) SD: 0.89 (mon)	M: 1.95 (mon.) SD: 0.84 (mon.)	Infant	English	Interaction	0.4903 (0.4903)
(Quigley et al. 2016) [52]	10	9	M: 8.27 (mon.) SD: 0.85 (mon.)	M: 8.13 (mon.) SD: 0.88 (mon.)	Infant	English	Interaction	0.8738 (0.4808)
(Maes et al., 2023) [57]	10	10	M: 4 (Yr.); 06.9 (mon.) SD: 1 (Yr.); 00.23 (mon.)	M: 4 (Yr.); 06.54 (mon.) SD: 0 (Yr.); 09.8 (mon.)	Children	French	Interaction	0.1603 (0.4479)
**Name**	**N_ASD**	**N_TD**	**Age_ASD**	**Age_TD**	**Group**	**Language**	**Task**	**RateASDvsTD**
(Ochi et al., 2019) [34]	65	17	M: 26.9 (Yr.) SD: 7.0 (Yr.)	M: 29.6 (Yr.) SD: 7.0 (Yr.)	Adult	Japanese	Interaction	−0.1743 (0.2728)
(Dahlgren et al., 2018) [29]	11	11	M: 11.1 (Yr.) SD: 1.10 (Yr.)	M: 11.1 (Yr.) SD: 0.47 (Yr.)	Children	NA	Narration	−0.1182 (0.4268)
(Cho et al., 2023) [15]	45	47	M: 25.7 (mon.) SD: 3.63 (mon.)	M: 25.5 (mon.) SD: 3.21 (mon.)	Chidlren	Chinese	Conversation	−0.33 (0.1464)
(Choi and Lee, 2019) [55]	17	34	M: 98.8 (mon.) SD: 18.6 (mon.)	M: 99.3 (mon.) SD: 20.7 (mon.)	Children	Korean	Conversation	0.2399 (0.298)
(Nadig and Shaw, 2012) [3]	15	13	M: 11.0 (Yr.) SD: 19 (mon.)	M: 11.0 (Yr.) SD: 24 (mon.)	Children	English	Conversation	0.5177 (0.3852)
(Nadig and Shaw, 2012) [3]	15	11	M: 10.6 (Yr.) SD: 17 (mon.)	M: 10.8 (Yr.) SD: 23 (mon.)	Children	English	Interaction	0.0686 (0.397)

**Table 3:** Summary of machine learning characteristics in included studies.

Authos	Sample Size	Task	Performance
(Oller et al., 2010) [62]	ASD: 77; TD: 106	Interaction	ACC: 0.86; SENS: 0.75; SPEC: 0.98
(Kiss et al., 2012) [42]	ASD: 14; TD: 28	Interaction	AUC: 0.75; ACC: 0.74; SPEC: 0.57
(Fusaroli et al., 2013) [63]	ASD: 10; TD: 13	Narration	ACC: 0.86; SENS: 0.884; SPEC: 0.854
(Fusaroli, Grossman, et al., 2015) [64]	ASD: 52; TD: 34	Narration	ACC: 0.7165; SENS: 0.5832; SPEC: 0.8442
(Fusaroli, Grossman, et al., 2015) [64]	ASD: 26; TD: 34	Narration	ACC: 0.8201; SENS: 0.848; SPEC: 0.8139
(Fusaroli, Lambrechts, et al., 2015) [65]	ASD: 17; TD: 17	Narration	ACC: 0.819; SENS: 0.8483; SPEC: 0.822
(Asgari et al., 2021) [32]	ASD: 90; TD: 28	Conversation	AUC: 0.82; ACC: 0.733; SENS: 0.6967; SEPC: 0.7683
(Santos et al., 2013) [19]	ASD: 23; TD: 20	Conversation	AUC: 0.66; ACC: 0.628; SPEC: 0.55
(Santos et al., 2013) [19]	ASD: 23; TD: 20	Conversation	AUC: 0.97; ACC: 0.977; SPEC: 1
(MacFarlane et al., 2022) [39]	ASD: 88; TD: 70	Interaction	AUC: 0.78; ACC: 0.7215; SENS: 0.75; SPEC: 0.6857
(MacFarlane et al., 2022) [39]	ASD: 88; TD: 70	Interaction	AUC: 0.8748; ACC: 0.7975; SENS: 0.7727; SPEC: 0.8286
(MacFarlane et al., 2022) [39]	ASD: 88; TD: 70	Interaction	AUC: 0.9205; ACC: 0.8671; SENS: 0.8977; SPEC: 0.8266
(Lau et al., 2022) [5]	ASD: 83; TD: 63	Narration	AUC: 0.886; ACC: 0.835; SENS: 0.79; SPEC: 0.877
(Lau et al., 2022) [5]	ASD: 83; TD: 63	Narration	AUC: 0.559; ACC: 0.566; SENS: 0.632; SPEC: 0.509
(Rybner et al., 2022) [66]	ASD: 10; TD: 8	Narration	ACC: 0.89; SENS: 0.75; SPEC: 1; PREC: 1
(Rybner et al., 2022) [66]	ASD: 28; TD: 32	Narration	ACC: 0.68; SENS: 0.5; SPEC: 0.76; PREC: 0.82
(Plank et al., 2023) [60]	ASD: 26; TD: 54	Conversation	ACC: 0.762; SENS: 0.738; SPEC: 0.76; PREC: 0.63
(Chowdhury et al., 2023) [67]	ASD: 14; TD: 15	Conversation	ACC: 0.76; SENS: 0.64; SPEC: 0.87; PREC: 0.84

## Data Availability

The datasets generated during and/or analyzed during the current study are available from the corresponding author on reasonable request.

## References

[1] Patel S.P., Nayar K., Martin G.E., Franich K., Crawford S., Diehl J.J., Losh M. (2020). An Acoustic Characterization of Prosodic Differences in Autism Spectrum Disorder and First-Degree Relatives. J. Autism Dev. Disord..

[2] Redford M.A., Kapatsinski V., Cornell-Fabiano J. (2018). Lay Listener Classification and Evaluation of Typical and Atypical Children’s Speech. Lang. Speech.

[3] Nadig A., Shaw H. (2012). Acoustic and perceptual measurement of expressive prosody in high-functioning autism: Increased pitch range and what it means to listeners. J. Autism Dev. Disord..

[4] Bone D., Lee C.-C., Black M.P., Williams M.E., Lee S., Levitt P., Narayanan S. (2014). The psychologist as an interlocutor in autism spectrum disorder assessment: Insights from a study of spontaneous prosody. J. Speech Hear. Res..

[5] Lau J.C.Y., Patel S., Kang X., Nayar K., Martin G.E., Choy J., Wong P.C.M., Losh M. (2022). Cross-linguistic patterns of speech prosodic differences in autism: A machine learning study. PLoS ONE.

[6] Loveall S.J., Hawthorne K., Gaines M. (2021). A meta-analysis of prosody in autism, williams syndrome, and down syndrome. J. Commun. Disord..

[7] Chen S., Xiong J., Chen B., Zhang C., Deng X., He F., Yang L., Chen C., Peng J., Yin F. (2022). Autism spectrum disorder and comorbid neurodevelopmental disorders (ASD-NDDs): Clinical and genetic profile of a pediatric cohort. Clin. Chim. Acta.

[8] American Psychiatric Association (APA) (2013). Diagnostic and Statistical Manual of Mental Disorders.

[9] Robledo J., Donnellan A.M. (2016). Supportive Relationships in Autism Spectrum Disorder: Perspectives of Individuals with ASD and Supporters. Behav. Sci..

[10] Paul R., Augustyn A., Klin A., Volkmar F.R. (2005). Perception and production of prosody by speakers with autism spectrum disorders. J. Autism Dev. Disord..

[11] Shriberg L.D., Paul R., Black L.M., van Santen J.P. (2011). The hypothesis of apraxia of speech in children with autism spectrum disorder. J. Autism Dev. Disord..

[12] McCann J., Peppé S., Gibbon F., O’hare A., Rutherford M. (2007). Prosody and its relationship to language in school-aged children with high-functioning autism. Int. J. Lang. Commun. Disord..

[13] Chi N.A., Washington P., Kline A., Husic A., Hou C., He C., Dunlap K., Wall D.P. (2022). Classifying Autism from Crowdsourced Semistructured Speech Recordings: Machine Learning Model Comparison Study. JPP.

[14] Tager-Flusberg H. (2001). Understanding the language and communicative impairments in autism. Int. Rev. Res. Ment. Retard..

[15] Cho S., Cola M., Knox A., Pelella M.R., Russell A., Hauptmann A., Covello M., Cieri C., Liberman M., Schultz R.T. (2023). Sex differences in the temporal dynamics of autistic children’s natural conversations. Mol. Autism.

[16] Diehl J.J., Watson D., Bennetto L., Mcdonough J., Gunlogson C. (2009). An acoustic analysis of prosody in high-functioning autism. Appl. Psycholinguist..

[17] Drimalla H., Scheffer T., Landwehr N., Baskow I., Roepke S., Behnia B., Dziobek I. (2020). Towards the automatic detection of social biomarkers in autism spectrum disorder: Introducing the simulated interaction task (SIT). NPJ Digit. Med..

[18] Cho S., Liberman M., Ryant N., Cola M., Schultz R.T., Parish-Morris J. Automatic Detection of Autism Spectrum Disorder in Children Using Acoustic and Text Features from Brief Natural Conversations. Proceedings of the Interspeech.

[19] Santos J.F., Brosh N., Falk T.H., Zwaigenbaum L., Bryson S.E., Roberts W., Smith I.M., Szatmari P., Brian J.A. Very early detection of Autism Spectrum Disorders based on acoustic analysis of pre-verbal vocalizations of 18-month old toddlers. Proceedings of the 2013 IEEE International Conference on Acoustics, Speech and Signal Processing.

[20] Tanaka H., Sakti S., Neubig G., Toda T., Nakamura S. Linguistic and Acoustic Features for Automatic Identification of Autism Spectrum Disorders in Children’s Narrative. Proceedings of the Workshop on Computational Linguistics and Clinical Psychology: From Linguistic Signal to Clinical Reality.

[21] Asghari S.Z., Farashi S., Bashirian S., Jenabi E. (2021). Distinctive prosodic features of people with autism spectrum disorder: A systematic review and meta-analysis study. Sci. Rep..

[22] Fusaroli R., Lambrechts A., Bang D., Bowler D.M., Gaigg S.B. (2017). Is voice a marker for Autism spectrum disorder? A systematic review and meta-analysis. Autism Res..

[23] Li M., Tang D., Zeng J., Zhou T., Zhu H., Chen B., Zou X. (2019). An automated assessment framework for atypical prosody and stereotyped idiosyncratic phrases related to autism spectrum disorder. Comput. Speech Lang..

[24] Baron-Cohen S. (1995). Mind Blindness: An Essay on Autism and Theory of Mind.

[25] Ding H., Zhang Y. (2023). Speech Prosody in Mental Disorders. Annu. Rev. Linguist..

[26] Arciuli J., Arciuli J., Brock J. (2014). Prosody and autism. Communication in Autism.

[27] Shriberg L.D., Paul R., McSweeny J.L., Klin A.M., Cohen D.J., Volkmar F.R. (2001). Speech and prosody characteristics of adolescents and adults with high-functioning autism and Asperger syndrome. J. Speech Lang. Hear. R..

[28] Guo C., Chen F., Yan J., Gao X., Zhu M. (2022). Atypical prosodic realization by Mandarin-speaking autistic children: Evidence from tone sandhi and neutral tone. J. Commun. Disord..

[29] Dahlgren S., Sandberg A.D., Strömbergsson S., Wenhov L., Råstam M., Nettelbladt U. (2018). Prosodic traits in speech produced by children with autism spectrum disorders—Perceptual and acoustic measurements. Autism Dev. Lang. Impair..

[30] Hubbard D.J., Faso D.J., Assmann P.F., Sasson N.J. (2017). Production and perception of emotional prosody by adults with autism spectrum disorder. Autism Res..

[31] Brisson J., Martel K., Serres J., Sirois S., Adrien J.L. (2014). Acoustic analysis of oral productions of infants later diagnosed with autism and their mother. Infant Ment. Health J..

[32] Asgari M., Chen L., Fombonne E. (2021). Quantifying voice characteristics for detecting autism. Front. Psychol..

[33] Pepp’e S., McCann J., Gibbon F., O’Hare A., Rutherford M. (2007). Receptive and expressive prosodic ability in children with high-functioning autism. J. Speech Hear. Res..

[34] Ochi K., Ono N., Owada K., Kojima M., Kuroda M., Sagayama S., Yamasue H. (2019). Quantification of speech and synchrony in the conversation of adults with autism spectrum disorder. PLoS ONE.

[35] McCarty P., Frye R.E. (2020). Early Detection and Diagnosis of Autism Spectrum Disorder: Why Is It So Difficult?. Semin. Pediatr. Neurol..

[36] Kissine M., Geelhand P. (2019). Brief Report: Acoustic Evidence for Increased Articulatory Stability in the Speech of Adults with Autism Spectrum Disorder. J. Autism Dev. Disord..

[37] Bone D., Black M.P., Lee C.C., Williams M.E., Levitt P., Lee S., Narayanan S. Spontaneous-speech acoustic-prosodic features of children with autism and the interacting psychologist. Proceedings of the Interspeech.

[38] Bone D., Black M.P., Ramakrishna A., Grossman R.B., Narayanan S.S. Acoustic-prosodic correlates of ‘awkward’ prosody in story retellings from adolescents with autism. Proceedings of the Interspeech.

[39] MacFarlane H., Salem A.C., Chen L., Asgari M., Fombonne E. (2022). Combining voice and language features improves automated autism detection. Autism Res..

[40] Leightley D., Williamson V., Darby J., Fear N.T. (2018). Identifying probable post-traumatic stress disorder: Applying supervised machine learning to data from a UK military cohort. J. Ment. Health.

[41] Beccaria F., Gagliardi G., Kokkinakis D. Extraction and Classification of Acoustic Features from Italian Speaking Children with Autism Spectrum Disorders. Proceedings of the RaPID Workshop-Resources and Processing of Linguistic, Para-Linguistic and Extra-Linguistic Data from People with Various Forms of Cognitive/Psychiatric/Developmental Impairments-within the 13th Language Resources and Evaluation Conference.

[42] Kiss G., van Santen J.P.H., Prud’hommeaux E., Black L.M. Quantitative analysis of pitch in speech of children with neurodevelopmental disorders. Proceedings of the Interspeech.

[43] Kallay J.E., Dilley L., Redford M.A. (2022). Prosodic Development During the Early School-Age Years. J. Speech Lang. Hear. Res..

[44] Engstrand O. (1992). Systematicity of phonetic variation in natural discourse. Speech Commun..

[45] Furui S., Nakamura M., Ichiba T., Iwano K. (2005). Analysis and recognition of spontaneous speech using Corpus of Spontaneous Japanese. Speech Commun..

[46] Rischel J. (1992). Formal linguistics and real speech. Speech Commun..

[47] Jasmin K., Gotts S.J., Xu Y., Liu S., Riddell C.D., Ingeholm J.E., Kenworthy L., Wallace G.L., Braun A.R., Martin A. (2019). Overt social interaction and resting state in young adult males with autism: Core and contextual neural features. Brain.

[48] Hedges L.V. (1981). Distribution Theory for Glass’s Estimator of Effect Size and Related Estimators. J. Educ. Behav. Stat..

[49] Higgins J.P., Thompson S.G. (2002). Quantifying heterogeneity in a meta-analysis. Stat. Med..

[50] Doleman B., Freeman S., Lund J., Williams J., Sutton A. (2020). Identifying Publication Bias in Meta-Analyses of Continuous Outcomes in the Presence of Baseline Risk.

[51] Scharfstein L.A., Beidel D.C., Sims V.K., Rendon Finnell L. (2011). Social skills deficits and vocal characteristics of children with social phobia or Asperger’s disorder: A comparative study. J. Abnorm. Child Psychol..

[52] Quigley J., McNally S., Lawson S. (2016). Prosodic Patterns in Interaction of Low-Risk and at-Risk-of-Autism Spectrum Disorders Infants and Their Mothers at 12 and 18 Months. Lang. Learn. Dev..

[53] Pokorny F.B., Schuller B., Marschik P.B., Brueckner R., Nyström P., Cummins N., Bölte S., Einspieler C., Falck-Ytter T. Earlier Identification of Children with Autism Spectrum Disorder: An Automatic Vocalisation-Based Approach. Proceedings of the Interspeech 2017.

[54] Chan K.K., To C.K.S. (2016). Do Individuals with High-Functioning Autism Who Speak a Tone Language Show Intonation Deficits?. J. Autism Dev. Disord..

[55] Choi J., Lee Y. (2019). Conversational Factors Discriminating between High-Functioning Autism Spectrum Disorders and Typical Development: Perceptual Rating Scale. Commun. Sci. Disord..

[56] Sharda M., Subhadra T.P., Sahay S., Nagaraja C., Singh L., Mishra R., Sen A., Singhal N., Erickson D., Singh N.C. (2010). Sounds of melody-pitch patterns of speech in autism. Neurosci. Lett..

[57] Maes P., Weyland M., Kissine M. (2023). Structure and acoustics of the speech of verbal autistic preschoolers. J. Child Lang..

[58] Kaland C., Krahmer E.J., Swerts M. Contrastive intonation in autism: The effect of speaker- and listener-perspective. Proceedings of the Interspeech.

[59] Lehnert-LeHouillier H., Terrazas S., Sandoval S. (2020). Prosodic Entrainment in Conversations of Verbal Children and Teens on the Autism Spectrum. Front. Psychol..

[60] Plank I., Koehler J., Nelson A., Koutsouleris N., Falter-Wagner C. (2023). Automated extraction of speech and turn-taking parameters in autism allows for diagnostic classification using a multivariable prediction model. Front. Psychiatry.

[61] Morett L.M., O’Hearn K., Luna B., Ghuman A.S. (2016). Altered Gesture and Speech Production in ASD Detract from In-Person Communicative Quality. J. Autism Dev. Disord..

[62] Oller D.K., Niyogi P., Gray S., Richards J.A., Gilkerson J., Xu D., Yapanel U., Warren S.F. (2010). Automated vocal analysis of naturalistic recordings from children with autism, language delay, and typical development. Proc. Natl. Acad. Sci. USA.

[63] Fusaroli R., Bang D., Weed E. Non-Linear Analyses of Speech and Prosody in Asperger’s Syndrome. Proceedings of the IMFAR 2013.

[64] Fusaroli R., Grossman R.B., Cantio C., Bilenberg N., Weed E. The temporal structure of the autistic voice: A cross-linguistic examination. Proceedings of the IMFAR 2015.

[65] Fusaroli R., Lambrechts A., Yarrow K., Maras K., Gaigg S. Voice patterns in adult English speakers with Autism Spectrum Disorder. Proceedings of the IMFAR 2015.

[66] Rybner A., Jessen E.T., Mortensen M.D., Larsen S.N., Grossman R., Bilenberg N., Cantio C., Jepsen J.R.M., Weed E., Simonsen A. (2022). Vocal markers of autism: Assessing the generalizability of machine learning models. Autism Res..

[67] Chowdhury T., Romero V., Stent A. Parameter Selection for Analyzing Conversations with Autism Spectrum Disorder. Proceedings of the INTERSPEECH.

[68] Marchi E., Schuller B., Baron-Cohen S., Golan O., Bölte S., Arora P., Häb-Umbach R. Typicality and emotion in the voice of children with autism spectrum condition: Evidence across three languages. Proceedings of the Interspeech.

[69] Yarkoni T., Westfall J. (2017). Choosing prediction over explanation in psychology: Lessons from machine learning. Perspect. Psychol. Sci..

[70] Bishop C.M. (2006). Pattern Recognition and Machine Learning.

[71] Hastie T., Tibshirani R., Friedman J.H. (2009). The Elements of Statistical Learning: Data Mining, Inference, and Prediction.

[72] Arroabarren I., Carlosena A. Modelling of vibrato production. Proceedings of the 2004 12th European Signal Processing Conference.

[73] Lee J., Kim G.W., Kim S. (2021). Laryngeal height and voice characteristics in children with autism spectrum disorders. Phon. Speech Sci..

[74] Huttunen K.H., Keränen H.I., Pääkkönen R.J., Päivikki Eskelinen-Rönkä R., Leino T.K. (2011). Effect of cognitive load on articulation rate and formant frequencies during simulator flights. J. Acoust. Soc. Am..

[75] Thurber C., Tager-Flusberg H. (1993). Pauses in the narratives produced by autistic, mentally retarded, and normal children as an index of cognitive demand. J. Autism Dev. Disord..

[76] Arvaniti A. (2012). The usefulness of metrics in the quantification of speech rhythm. J. Phon..

[77] Grossman R.B., Bemis R.H., Plesa Skwerer D., Tager-Flusberg H. (2010). Lexical and affective prosody in children with high-functioning autism. J. Speech Lang. Hear. Res..

[78] Kanner L. (1943). Autistic disturbances of affective contact. Nerv. Child.

[79] Van Santen J.P.H., Prud’hommeaux E.T., Black L.M., Mitchell M. (2010). Computational prosodic markers for autism. Autism.

[80] Ye J. Rhythm theory. Proceedings of the Fifth National Conference on Modern Phonetics, Tsinghua University.

[81] Fine J., Bartolucci G., Ginsberg G., Szatmari P. (1991). The use of intonation to communicate in pervasive developmental disorders. J. Child Psychol. Psychiatry.

